# Numerical Simulation and Process Optimization on Hot Twist-Stretch Straightening of Ti-6Al-4V Alloy Profile

**DOI:** 10.3390/ma15134522

**Published:** 2022-06-27

**Authors:** Xuwen Deng, Songxiao Hui, Wenjun Ye, Rui Liu, Liang Huang

**Affiliations:** 1 State Key Laboratory of Nonferrous Metals and Processes, GRINM Group Co., Ltd., Beijing 100088, China; dengxuwen19950901@outlook.com (X.D.); huisx@grinm.com (S.H.); h_o_n_o_r@163.com (R.L.); 2 GRIMAT Engineering Institute Co., Ltd., Beijing 101407, China; 3 General Research Institute for Nonferrous Metals, Beijing 100088, China; 4 State Key Laboratory of Materials Processing and Die & Mould Technology, School of Materials Science and Engineering, Huazhong University of Science and Technology, Wuhan 430074, China; huangliang@hust.edu.cn

**Keywords:** Ti-6Al-4V profile, hot twist-stretch straightening, orthogonal experiment, thermo-mechanical coupling model

## Abstract

Ti-6Al-4V profiles prepared by hot extrusion are usually accompanied by bending and twisting. The hot twist-stretch straightening is an effective strategy such that the bending deflection and twisting angle can be simultaneously decreased by a single straightening process. In addition, utilizing stress relaxation effect, the residual stress and springback can be greatly reduced by holding the straightening temperature and strain constant for a period after twist-stretch straightening. In this study, the hot deformation behaviors of the Ti-6Al-4V profile were revealed by experiments. The tensile model was obtained by uniaxial tensile tests within ranges of temperatures (500–700 °C) and strain rates (5 × 10^−5^–1 × 10^−3^ s^−1^). The creep constitutive model was acquired with stress relaxation experiments in ranges of temperatures (500–700 °C) and pre-strain of 1.5%. Then, the coupled thermo-mechanical model of hot twist-stretch straightening was established. Based on orthogonal experiment strategy, the effects of straightening temperature, stretch strain, and holding time on the bending deflection and torsion angle of profile were investigated systematically and the process was optimized. The straightening accuracy is significantly affected by straightening temperature and holding time. By using optimized process parameters in practical straightening experiments, the deflection/length and angle/length after straightening does not exceed 2‰ and 2.5‰°/mm, respectively, which is basically consistent with the numerical simulation result.

## 1. Introduction

Ti-6Al-4V alloy, with the advantages of an excellent strength to weight ratio, corrosion resistance, and composite compatibility, is the most commonly used titanium alloy [1,2]. Its profiles have been applied to important structural components in large aircrafts such as beams and stringers [3,4]. At present, hot extrusion is the main production method of titanium alloy profile due to its high production efficiency and high material utilization [5]. However, the features of hot extrusion for titanium alloy profile are high extrusion temperature (950–115 °C [6]), fast extrusion rate (≥200 mm/s [7]), and the extrusion end cannot be pulled. In addition, the section shapes of the profile are complicated and multiplicate, thus causing the metal flow velocity in different regions to be inconsistent during hot extrusion [8]. These above reasons lead to profile products being inevitably accompanied by bending and twisting deformations [9]. The straightness and torsion of the profile will directly influence the subsequent machining quality and assembly accuracy. The twist-stretch straightening is an effective method to improve the compound deformations of the profile after extrusion, and can be applied to the profile with various section shapes, which has a high production flexibility and efficiency.

Ti-6Al-4V alloy has high strength and low elastic modulus, which is difficult to be formed by conventional cold processing [10,11]. Therefore, Ti-6Al-4V profiles are usually straightened in hot conditions. The straightening procedure for Ti-6Al-4V profile contain the following processes: firstly, the profile is heated to the desired temperature assisted by resistance heating. Subsequently, the profile is twisted and stretched in order to improve its straightness and torsion. Then, the straightening temperature and twist-stretch strain are maintained constant for a period to reduce internal stress. This process is also called creep forming. Finally, the profile is cooled to room temperature and unloaded. Compared to the conventional processing methods, creep forming has lots of advantages such as low residual stress, small springback, and high forming stability, which is widely utilized in the processing of titanium alloy [12]. Astarita et al. [13,14] established a hot stretch bending FE model of the Ti-6Al-4V profile, and measured the residual stress distribution by the hole-drilling method. The stress was significantly reduced and homogenized after creep forming. Deng et al. [15,16] established the constitutive modeling of OT4 and Ti-6Al-4V alloy, then created the hot stretch bending and creep forming FE model of the L profile. The stress distribution and deformation springback of the profile were predicted and verified. Guo et al. [17] considered heat conduction between the profile and die: the heat transfer coefficients were determined by inverse analysis and the non-isothermal FE model for hot stretch bending of the Ti-6Al-4V profile was established. The model indicated that temperature distribution would affect the springback value, improve the temperature of the die, and the profile could reduce springback. Zhao et al. [18] researched the bending creep process of Ti-6Al-4V L-shaped profile, and found that stress relaxation effect is mainly affected by temperature, with the residual stress after stress relaxation reduced to below 50 MPa under 700 °C. According to the above references, much research focused on the numerical simulations of hot bending and creep forming of the titanium alloy profile while little research has been reported on the straightening process.

The straightening process of the profile aims to improve the straightness while not changing its microstructure and mechanical property in an obvious way. The straightening temperature is commonly below annealing temperature, which ranges from 500 to 700 °C [18]. The holding time should not be too long, otherwise the production efficiency will be reduced and the surface oxidation will be intensified. The holding time should not exceed 1200 s [17]. Zhang et al. [19] studied the pre-stretching process of a large aluminum alloy sheet after quenching; the flatness could be significantly improved and most residual stress could be reduced after 1–3% stretch strain. Similarly, the stretch strain of the profile is generally set to be 1 to 2%. In summary, the hot twist-stretch straightening of the Ti-6Al-4V profile is a complex process controlled by multiple parameters. In order to improve the straightness and torsion of the profile so that they satisfy the assembly accuracy, optimization of the process on hot twist-stretch straightening of the Ti-6Al-4V alloy profile needs being researched urgently.

This paper mainly focused on the hot twist-stretch straightening of the Ti-6Al-4V alloy U-shaped profile. The tensile property and creep behavior of the profile under different conditions were obtained by uniaxial tensile tests and stress relaxation tests, respectively. Then, the coupled thermo-mechanical numerical simulation model was established in ABAQUS software. The simulation procedure includes four steps: twisting, stretching, holding, and springback. According to the orthogonal experiment strategy, the effect of straightening temperature, stretch strain, and holding time on the deflection and angle after straightening was researched systematically. Considering the simulation results and practical process state, the straightening process parameters of the profile were optimized. Finally, the verification experiments were carried out to validate the accuracy of the numerical simulation model. 

## 2. Material and Methods

### 2.1. Experiment Material

**Figure 1:**
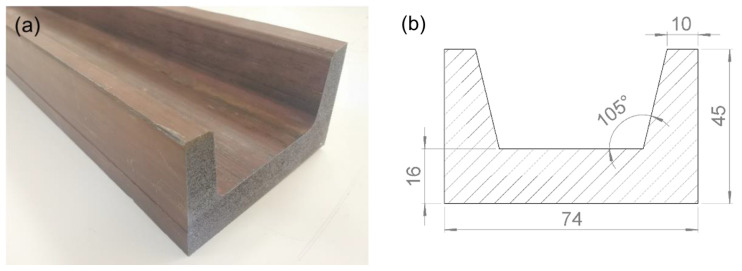
Material for experiments: (**a**) prepared profile; (**b**) section size (mm).

**Figure 2:**
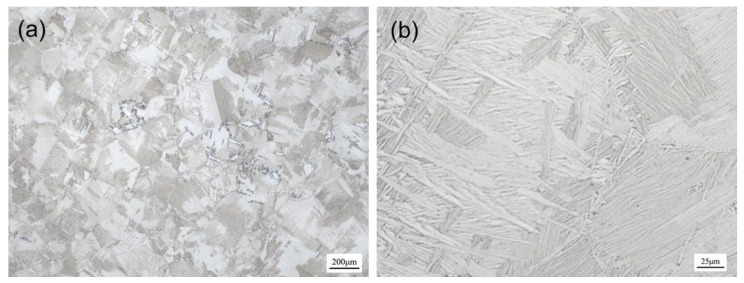
Microstructure of extruded Ti-6Al-4V alloy profile: (**a**) 50×; (**b**) 500×.

The research object in this study was a Ti-6Al-4V alloy thick-walled profile with a U-shaped section. The length of the profile was 1 m. The profile was prepared by hot extrusion with an extrusion temperature of 1150 °C, extrusion ratio of 25, and extrusion speed of 200 mm/s. The prepared profile is shown in Figure 1a and the section size is shown in Figure 1b. The chemical composition of the profile is listed in Table 1. The microstructure of the profile is a Widmanstätten structure with lamellar α colonies in coarse β grains of about 200 μm, as presented in Figure 2a,b.

### 2.2. Uniaxial Tensile Tests

**Figure 3:**
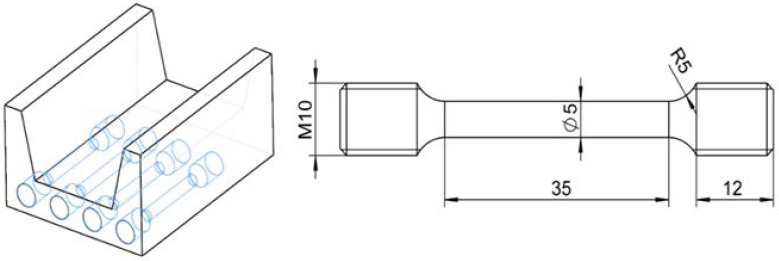
Schematic of uniaxial tensile test specimen (mm).

The material property is the foundation for establishing a numerical simulation model; therefore, it is needed to carry out a series of experiments in order to obtain the material property of the Ti-6Al-4V profile. The twisting and stretching processes can be described by tensile properties acquired by performing uniaxial tensile tests. The uniaxial tensile tests were performed on MTS universal testing machine equipped with a heating furnace. The specimens were taken from the length direction of the profile; the sampling position and dimension of the specimens are presented in Figure 3. The specimens were heated to the deformation temperature and held for a period to ensure the uniformity of the temperature. The tests were performed at temperatures of 500, 600, and 700 °C and the strain rates of 5 × 10^−5^, 1 × 10^−4^, 5 × 10^−4^, and 1 × 10^−3^ s^−1^. The strain of specimen was recorded by an extensometer with a standard distance of 25 mm.

### 2.3. Stress Relaxation Tests

**Figure 4:**
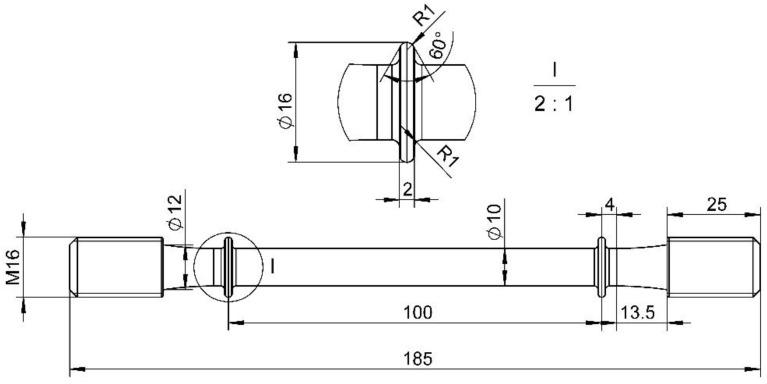
Dimension of stress relaxation specimen (mm).

The holding process during straightening is essentially consistent with stress relaxation. Thus, the deformation behavior during the holding period can be revealed by stress relaxation tests. The stress relaxation tests were performed on an RD-50 electronic creep testing machine. The sampling position of stress relaxation specimens were consistent with uniaxial tensile specimens; the dimension is displayed in Figure 4. The specimens were heated to experiment temperature and then pre-stretched to the setting strain. Subsequently, the strain was held constant and recorded the stress varying with time. The experiment temperature ranged from 500–700 °C, the pre-strain was 1.5%, and the relaxation time was 2000 s.

## 3. Numerical Simulation Model

### 3.1. Material Model

#### 3.1.1. Uniaxial Tensile Test Results

**Figure 5:**
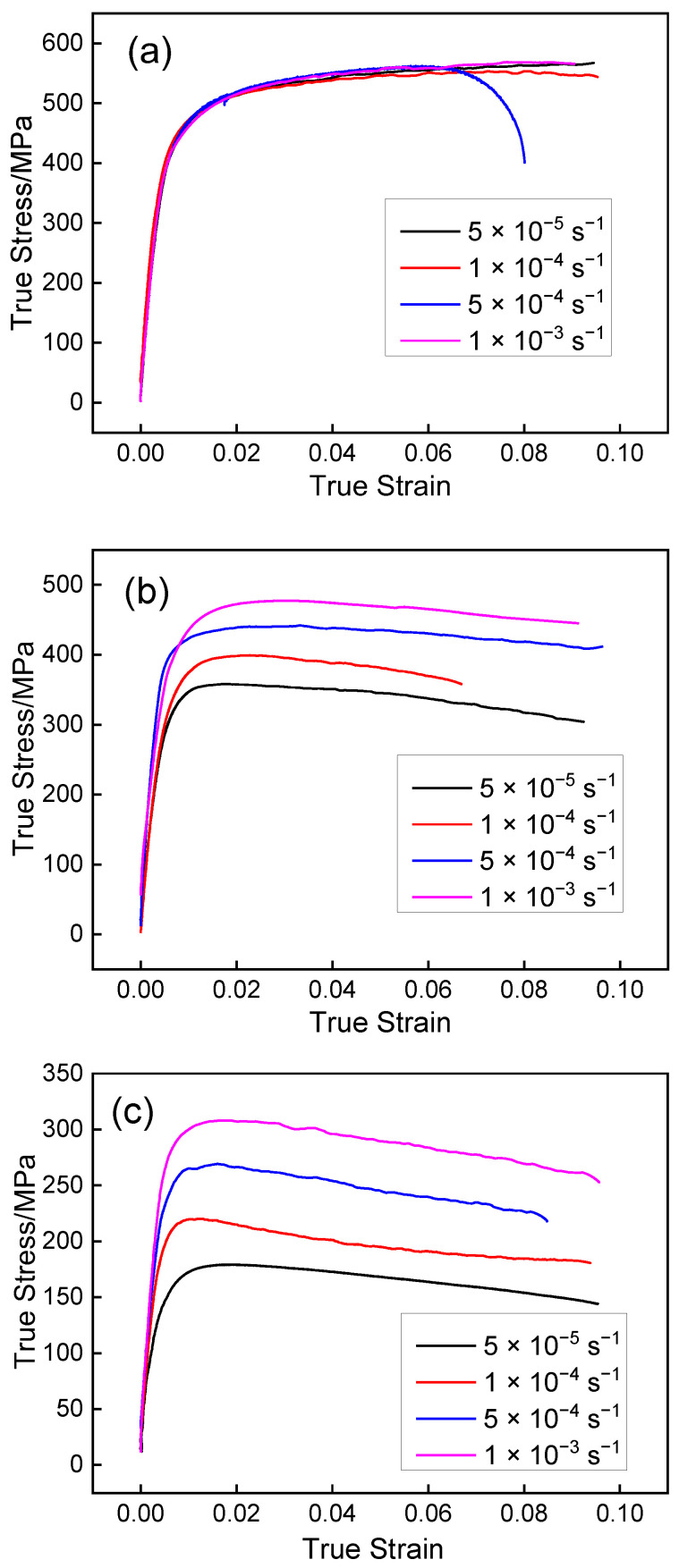
Flow stress curves of tensile specimens: (**a**) 500 °C; (**b**) 600 °C; (**c**) 700 °C.

Figure 5 shows the flow stress curves of tensile specimens at different temperatures and strain rates. Flow stress is mainly influenced by hardening and softening behavior which depends on temperature and strain rate [20,21,22]. It can be seen that flow stress decreases clearly as the temperature increases. With the increase of the deformation temperature, partial α phase transforms into β phase; the increasing proportion of β phase is conducive to the plastic deformation of titanium alloy [23]. At the temperature of 500 °C, the deformation mechanism is dominated by strain hardening, where the flow stress increases as the strain increases, and there was no discernible effect of strain rate on flow stress. By increasing the temperature to 600 °C and above, the flow stress increases with the strain rate increase. The curves present a peak stress at a certain strain, which is followed by dynamic softening. The tensile property of the Ti-6Al-4V profile can be defined by the multi-segments method directly in ABAQUS software.

#### 3.1.2. Stress Relaxation Test Results

**Figure 6:**
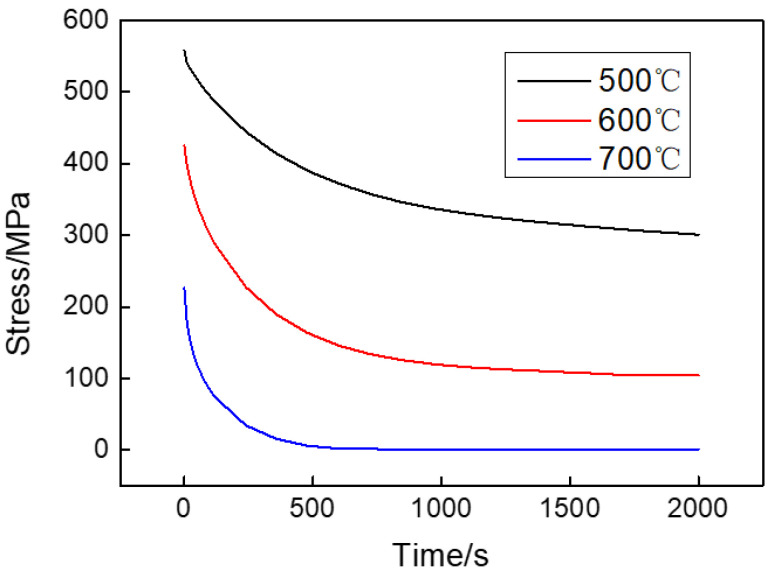
Stress relaxation curves of creep specimens.

The stress relaxation curves at different temperatures are presented in Figure 6. It can be seen that the stress variation contains two stages: at stage I, the stress decreases rapidly as time increases; at stage II, the decreasing rate of stress is very slow and the stress almost keeps constant. The higher the temperature is, the higher the stress relaxation rate and the smaller the relaxation limit stress.

#### 3.1.3. Creep Constitutive Modeling

The stress relaxation curves cannot directly import to finite element software, so the constitutive model needs to be calculated based on experimental results and then imported. There is an inherent unity between stress relaxation and creep; only the manifestations create differences [24]. Therefore, the stress relaxation behavior can be described through the creep model. In this study, the Zener–Hollomon parameter and the Arrhenius constitutive model were used to define the creep behavior of the Ti-6Al-4V profile. The model is expressed as follows [25,26,27]:

(1) $$Z=\dot{\varepsilon_{c}}\exp\left(\frac{Q}{RT}\right)$$

(2) $$\dot{\varepsilon_{c}}=AF\left(\sigma \right)\exp\left(-\frac{Q}{RT}\right)$$

(3) $$F\left(\sigma \right)=\{\begin{array}{ll} \sigma^{n_{1}} & \alpha \sigma <0.8\\ \exp\left(\beta \sigma \right) & \alpha \sigma >1.2\\ \sin h{\left(\alpha \sigma \right)}^{n} & \;\mathrm{for}\;\mathrm{all}\;\sigma \end{array}$$

where $\dot{\varepsilon_{c}}$ is the creep strain rate (s^−1^); *R* is the universal gas constant (8.314 J·K/mol); *Q* is the activation energy of hot deformation (J/mol); *T* is the temperature (K); σ is the flow stress (MPa); *A*, *n*_1_, *n*, *α*, *β* are parameters needed to be fitted.

During the whole stress relaxation process, there exists the following equation:

(4) $$\varepsilon_{t}=\varepsilon_{c}+\varepsilon_{e}$$

where $\varepsilon_{t}$ is the total strain; $\varepsilon_{c}$ is the creep strain; and $\varepsilon_{e}$ is the elastic strain. 

The characteristics of the stress relaxation process are that the total strain remains at zero consistently, and the elastic strain gradually transforms into the creep strain. By taking the derivation of time in Equation (4), Equation (5) can be obtained as follows:

(5) $$\dot{\varepsilon_{c}}=\frac{d\varepsilon_{c}}{dt}=-\frac{d\varepsilon_{e}}{dt}=-\frac{1}{E}\frac{d\sigma }{dt}$$

where *E* is the elastic modulus; dσ*/dt* is the first derivative of stress to time. By uniting Equation (5) and the stress relaxation curves in Figure 6, the relationship between $\dot{\varepsilon_{c}}$ and σ can be obtained.

Taking the natural logarithm of Equation (2) when ασ<0.8, the following equation can be acquired:

(6) $$\ln\dot{\varepsilon_{c}}=n_{1}\ln\sigma +A_{1}$$

**Figure 7:**
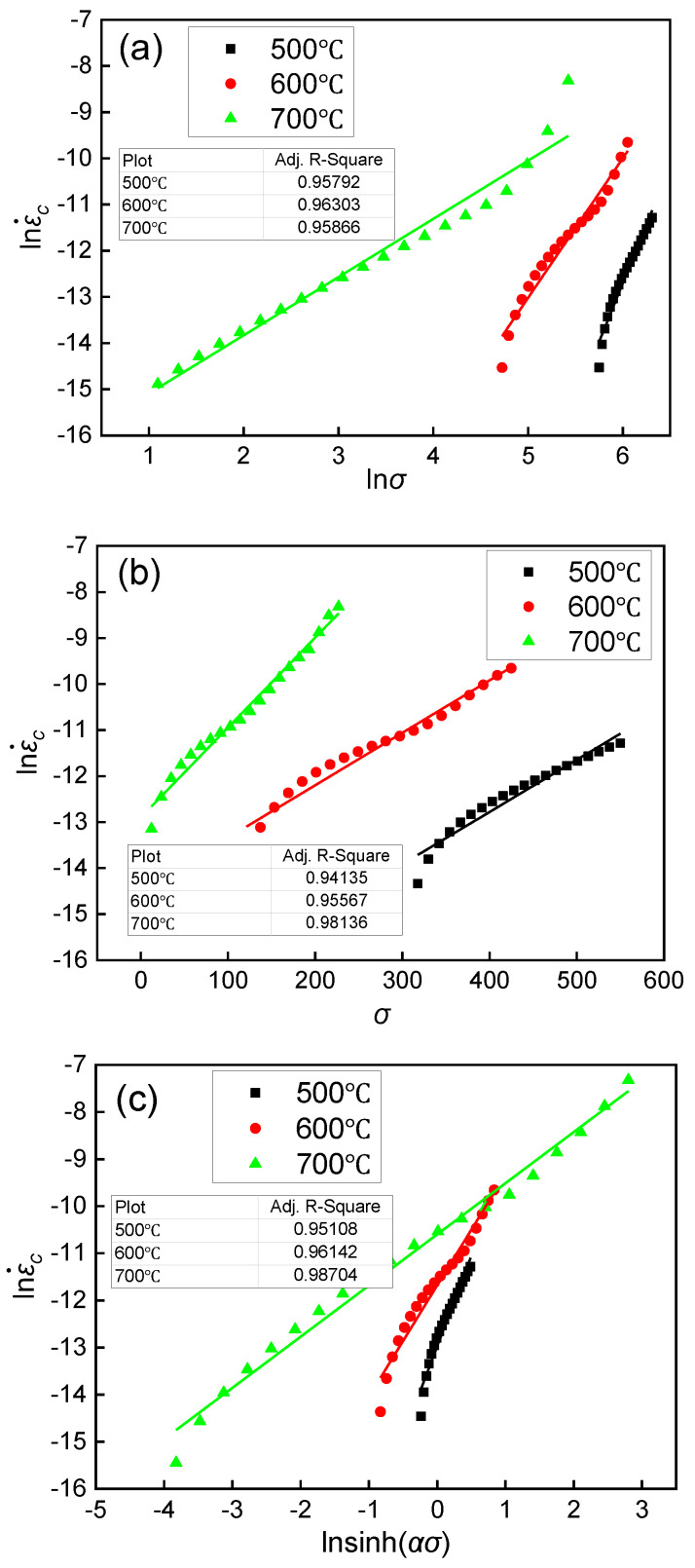
Linear fitting results of the constitutive model: (**a**) $\ln\dot{\varepsilon_{c}}–\ln\sigma$; (**b**) $\ln\dot{\varepsilon_{c}}–\sigma$; (**c**) $\ln\dot{\varepsilon_{c}}–\mathrm{lnsinh}\left(\alpha \sigma \right)$.

Then, the value of *n*_1_ under different temperatures can be obtained from the slope of the relationship between ln$\dot{\varepsilon_{c}}$ and lnσ, as shown in Figure 7a.

By taking the natural logarithm of Equation (2) when ασ>1.2, Equation (7) can be gained as follows:

(7) $$\ln\dot{\varepsilon_{c}}=\beta \sigma +\ln A_{2}-\frac{Q}{RT}$$

The linear fitting of $\ln\dot{\varepsilon_{c}}$ and *σ* under different temperatures was performed based on Equation (7), as shown in Figure 7b. The value of *β* could be obtained from the slope. Then, the value of α was calculated by *β = αn*_1_.

In the range of all *σ*, the following equation can be obtained by taking the natural logarithm of Equation (2):

(8) $$\ln\dot{\varepsilon_{c}}=n\mathrm{lnsinh}\left(\alpha \sigma \right)+\ln A-\frac{Q}{RT}$$

The fitting results of $\ln\dot{\varepsilon_{c}}$ and lnsinh(ασ) are shown in Figure 7c. The values of *n*, ln*A*, and *Q* can be obtained from the y-intercept and the slope.

In summary, the fitted parameters of the Arrhenius constitutive model are shown in Table 2.

**Figure 8:**
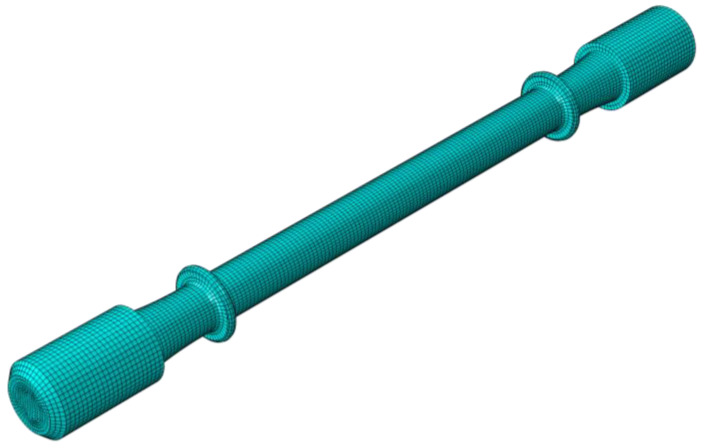
Meshed model of the creep specimen.

**Figure 9:**
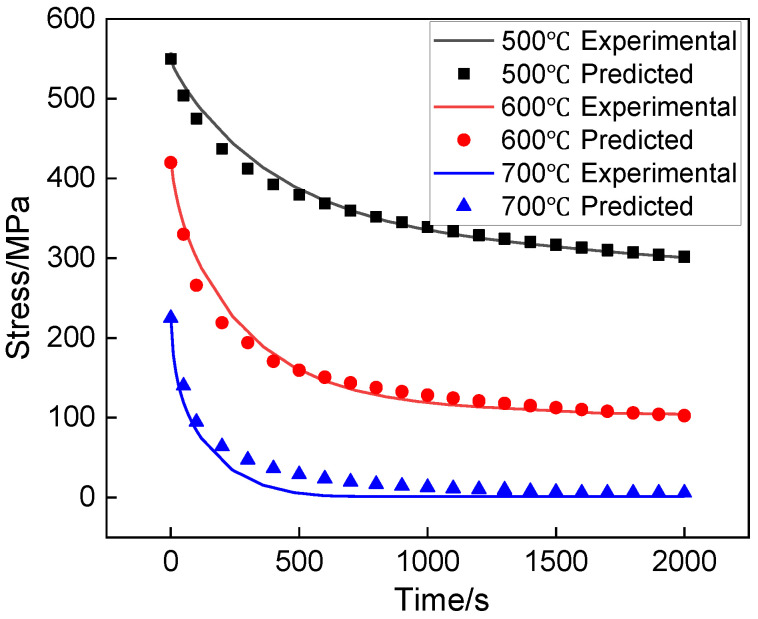
Comparisons between experimental and predicted results.

In order to verify the accuracy of the Arrhenius constitutive model, the numerical simulation model for the stress relaxation process of the creep specimen was established. The meshed model of the creep specimen is shown in Figure 8. The stress relaxation curve comparisons between the experimental and predicted data are shown in Figure 9. It can be seen that the results are identical, and the R^2^ values between the simulated and predicted curves under the temperatures of 500, 600, and 700 °C are 0.983, 0.973, and 0.935, respectively. This indicates that the fitted constitutive model is feasible for describing the creep behavior of the Ti-6Al-4V alloy profile.

### 3.2. Geometric Model

**Figure 10:**
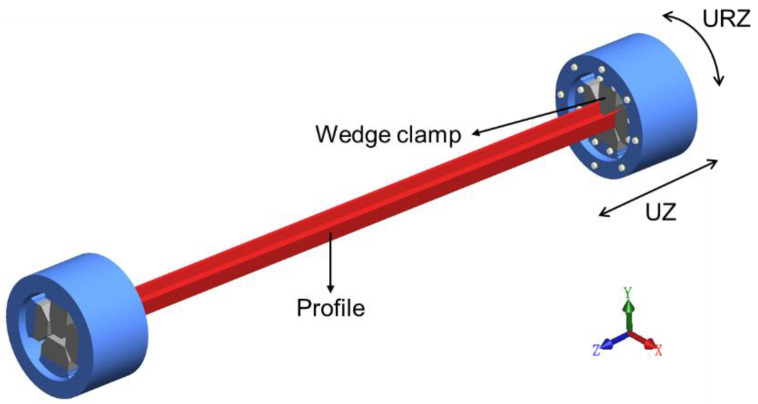
The hot straightening equipment model of the profile.

**Figure 11:**
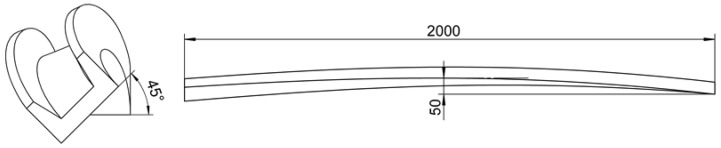
The geometric model of the initial shape of the profile.

The intact geometric model for profile straightening is presented in Figure 10, which includes the profile, wedge clamps, and a hydraulic twisting-stretching device. The clamping end could execute deformations of two degrees of freedom: UZ and URZ. To facilitate research, only the geometric model of the profile was established for numerical simulation while the heat conduction and contact between clamps and profile was ignored. The length and shape of the profile to be straightening were commonly irregular. The shapes of the profile were set to be uniform compound deformations with bending and twisting in this study. The profile’s length, bending deflection, and twisting angle were 2000 mm, 50 mm, and 45°, respectively, as shown in Figure 11.

### 3.3. Coupled Thermo-Mechanical Model

**Figure 12:**
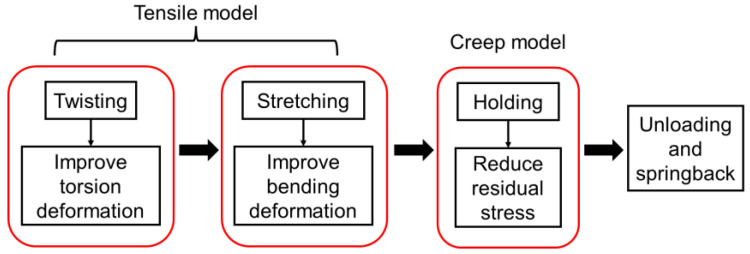
The flow chart of the numerical simulation procedure.

**Figure 13:**
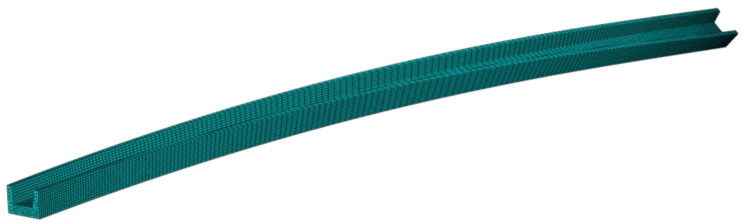
Meshed model of the profile to be straightened.

The numerical simulation model of hot twist-stretch straightening was established in ABAQUS software. The procedure was divided into four steps: twisting, stretching, holding, and springback. The step sequence and corresponding material model are shown in Figure 12. The profile was set as 8-node reduced integration thermally coupled elements (C3D8RT). The element size on the section was set to be 4 mm uniformly. The elements on the length direction of the profile were meshed with a higher density at both ends and a lower density at the middle region, with the sizes ranging from 4–20 mm. The meshed model is shown in Figure 13. The tensile and stress relaxation properties of the Ti-6Al-4V profile were presented in Section 3.1. The coupled temp-disp type was used in the twisting, stretching, and springback steps. The visco type was used in the holding step. The initial deformation and stress field of each step were inherited from the result file of previous step, successively. In addition, the profile was subjected to a stress relief annealing process before straightening, so the initial stress of the profile can be assumed to be 0 MPa.

### 3.4. Orthogonal Experiment Scheme

There are many parameters which influence the straightness and torsion of the profile during the straightening process, such as straightening temperature, stretch strain, twist angle, and holding time. In this study, the effects of temperature, stretch strain, and holding time on straightening were discussed and optimized by numerical simulation. The twist angle was consistent in value and opposite in direction with the initial torsion angle of the profile. According to the practical straightening process state, the orthogonal experiment was designed, and the influencing factors and levels are listed in Table 3. The bending deflection and torsion angle after straightening were taken as indicators for process optimization.

## 4. Results and Discussion

### 4.1. Discussion of Numerical Simulation Results

**Figure 14:**
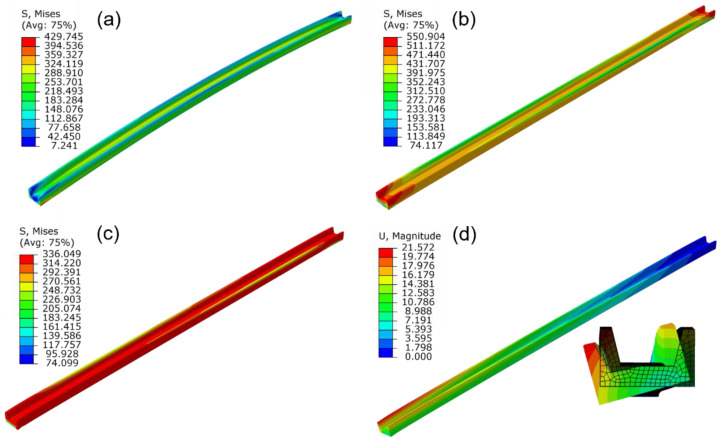
The simulation diagram of the straightening procedure: (**a**) twisting; (**b**) stretching; (**c**) holding; (**d**) springback.

The deformation contours of the profile after different straightening steps are shown in Figure 14. The shear stress occurred in the profile and the torsion angle of the profile was significantly decreased during the twisting process, as shown in Figure 14a. Then, followed by stretching, the straightness of the profile was improved obviously, but there still exists a large internal stress in the profile. During the holding process, the elastic deformation of the profile transforms into plastic deformation gradually as time increases due to the stress relaxation effect. The stress after stress relaxation (Figure 14c) was significantly reduced compared to that before (Figure 14b). Finally, the profile was cooled to room temperature and unloaded. After unloading, the profile would form a certain level of elastic springback along the initial directions of bending and twisting, as shown in Figure 14d.

**Figure 15:**
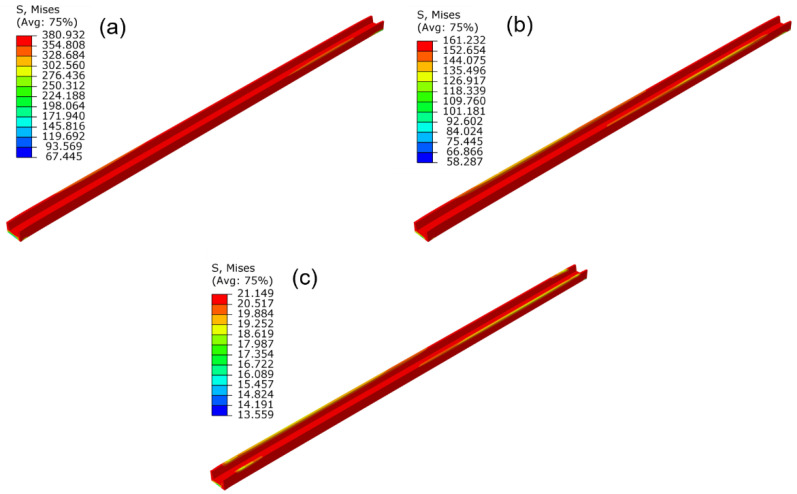
The influence of the straightening temperature on the stress distribution of the profile: (**a**) 500 °C; (**b**) 600 °C; (**c**) 700 °C (stretch strain 1.5%, holding time 600 s).

The stress contours of the profile under different straightening temperatures are shown in Figure 15. It could be concluded that the stress distribution in different regions of the profile is basically uniform. The straightening temperature directly affects the stress value of the profile, the flow stress was lower, and the stress relaxation effect was more evident under a higher straightening temperature. In addition, a higher stress level would result in a larger springback value after unloading. Therefore, with an increase in the straightening temperature, the stress after straightening decreases, and the straightness of the profile improves gradually. The torsion angle variation with the temperature has the same law as the bending deformation. The shear stress on the section of the profile was also reduced with the increase in the straightening temperature, and the torsion angle was gradually reduced.

**Figure 16:**
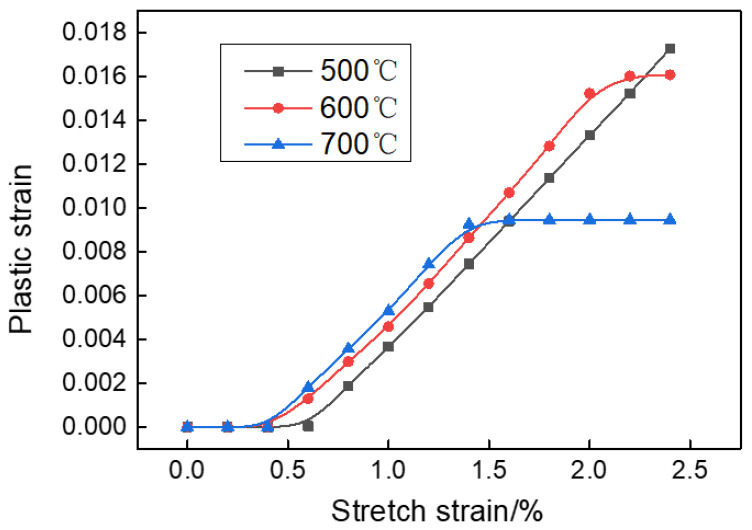
The relationship between plastic strain and stretch strain.

The stretch strain mainly affects the plastic strain of the profile. Generally, the larger the plastic strain is, the more sufficient the deformation and the better the straightening improvement. The curves of the plastic strain varying with the stretch strain are shown in Figure 16. The plastic strain-stretch strain curves consist of three stages. At stage I: only elastic deformation occurred in the profile so that the plastic strain was 0. The range of the elastic deformation area decreased as the temperature increased. At stage II, there was a linear increase correlation between the plastic strain and the stretch strain, approximately. At stage III, the plastic strain almost remained constant. The start points of stage III corresponded to the peak point in the flow stress curve under each temperature. According to the discussion in Section 3.1.1, the deformation mechanism of the profile is dominated by dynamic softening at the deformation temperature of 600 or 700 °C. When stretch strain exceeds the strain of the peak point, the flow stress decreases with the increase in strain, and the local necking has occurred in the profile at this stage. If the stretch strain continues to increase, only the plastic strain of the local necking region gradually increases while the other region remains constant.

**Figure 17:**
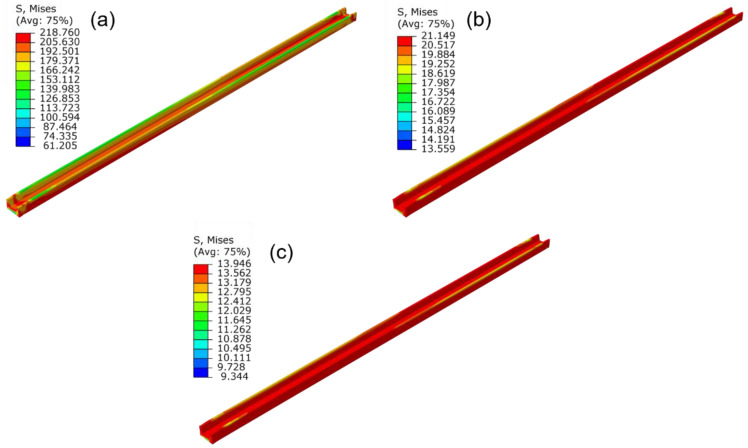
Influence of holding time on the stress distribution of the profile: (**a**) 0 s; (**b**) 600 s; (**c**) 1200 s (straightening temperature 700 °C, stretch strain 1.5%).

The stress contours of the profile under different holding time are presented in Figure 17. It can be seen that the Mises stress decreases with the increase in holding time. The maximum Mises stress decreases from 219 to 21 MPa when the holding time increases from 0 to 600 s, with the relative decreasing amplitude as large as 90%. Furthermore, the difference between the maximum and minimum stress of the profile decreases from 158 to 8 MPa, which indicates that the stress distribution was significantly homogeneous after stress relaxation. When the holding time increased from 600 to 1200 s, the stress decreasing amplitude was only 7 MPa. This result indicated that stress relaxation effect becomes inapparent as the holding time increases.

**Figure 18:**
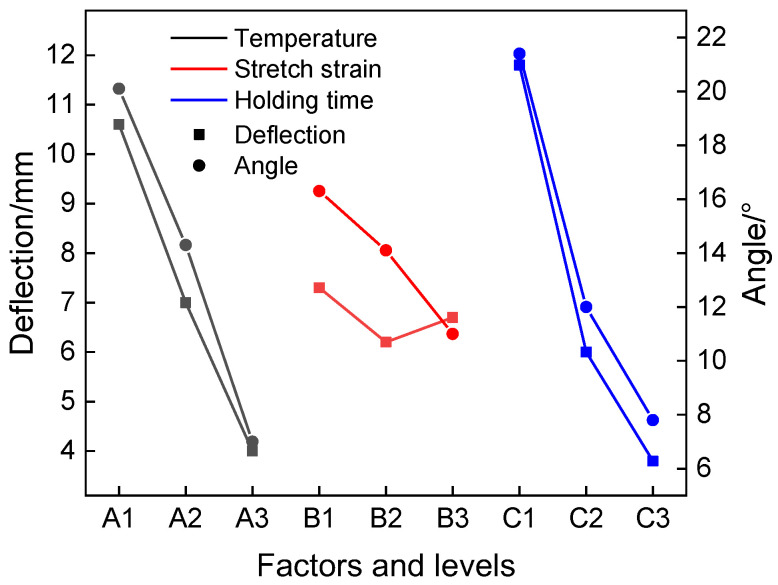
The deflection and angle corresponding to different factors and levels.

According to the numerical simulation results, the deflection and angle of profile after straightening under different orthogonal experiment parameters are listed in Table 4. It can be seen that R(A) = 5.9 mm/13.1°, R(B) = 1.1 mm/5.3°, R(C) = 8 mm/13.6°; the larger value of R means a larger variation range of deflection and angle under different levels, which indicates that the corresponding process parameter has great influence on straightening. Therefore, the straightening temperature and holding time are important factors affecting the deflection and angle of profile, while the influence of the strain is relatively small. With changes of different factors and levels, the variation of deflection and angle are shown in Figure 18. It can be seen that with the increase of straightening temperature (A1 to A3), the deflection of the profile decreased continuously due to the decrease of stress. With the increase of holding time (C1 to C3), the straightness of the profile improved obviously in the first 600 s and became less evident in the second 600 s, which is because stress relaxation effect becomes inapparent. The variation trends of the angle with the straightening temperature and holding time are consistent with those of deflection. However, the effect of stretch strain on deflection and angle was different: with the increase of stretch strain (B1 to B3), the angle decreased continuously while the deflection decreased firstly and then increased. This could be attributed to the fact that when the stretch strain exceeded the strain of the peak point in the flow stress curve, local necking occurred in the profile due to dynamic softening effect. Increasing the stretch strain has no significant benefit to improving the straightness.

When the deflection and angle reach a minimum value under the influence of various factors, the corresponding level is the optimal process parameter. With the discussion of Figure 18, the combination of the straightening temperature of 700 °C (A3), a strain of 1.5% (B2), and a holding time of 1200 s (C3) was the best process parameter. However, when the holding time increased from 600 to 1200 s under the straightening temperature of 700 °C, the stress relaxation effect became inapparent according to the results in Figure 17. Considering the practical straightening production efficiency, A3B2C2 was the optimum process parameter combination. The profile under this straightening process combination has a bending deflection(mm)/length(mm) of 1.5‰ and torsion angle(°)/length(mm) of 1.7‰°/mm.

### 4.2. Experimental Verification

The straightening experiments were carried on the 100 t stretch straightening machine. Firstly, the profile was loaded into the straightening machine and pre-tightened at room temperature. Then, the profile was heated to 700 °C assisted by resistance heating. When it reached setting temperature, the profile was straightened through twisting and stretching. The twisting angle was consistent in value and opposite in direction with the initial angle of the profile, and the stretching strain was 1.5%. Subsequently, the straightening temperature and twist-stretch strain of the profile were kept constant for 600 s in order to reduce internal stress. Finally, the profile was cooled to room temperature by controlling the cooling rate to 30–50 °C/min and unloaded. The two profiles were straightened to verify the accuracy of the numerical simulation model. The straightening results are listed in Table 5, and compared to the numerical simulation results, the maximum differences of deflection/length and angle/length were 0.7‰ and 0.5‰°/mm.

**Figure 19:**
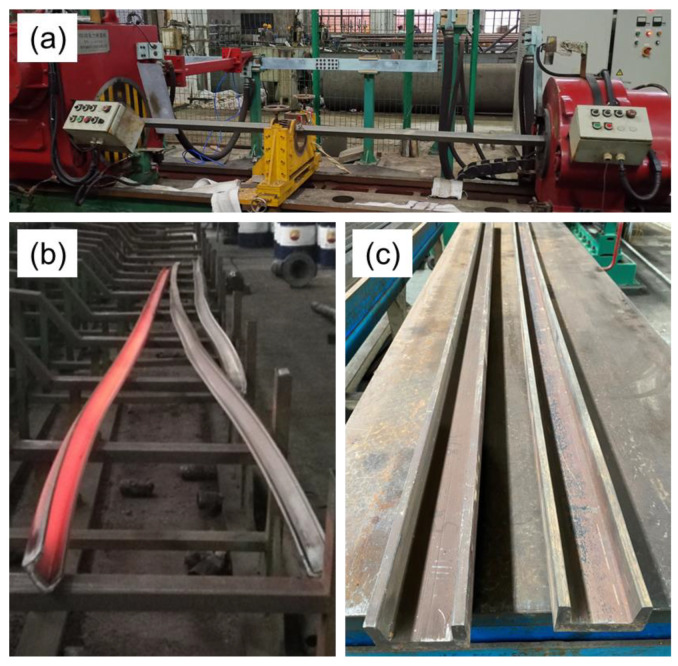
Industry production of the straightening process: (**a**) schematic diagram of straightening; (**b**) profiles before straightening; (**c**) profiles after straightening.

By applying the optimized straightening processes to practical production, profiles can achieve excellent flatness with high production stability. The schematic diagram of the straightening process for titanium alloy profiles is shown in Figure 19a. The comparison of profiles before and after straightening are presented in Figure 19b,c. It can be seen that the shapes of profiles prepared by hot extrusion were irregular: different profiles all have a certain degree of bending and twisting deformations. Profiles before straightening commonly had deflection(mm)/length(mm) values ranging from 5–50‰ and angle(°)/length(mm) values ranging from 10–40°/‰. After straightening, the straightness and torsion of profiles were improved significantly. The deflection/length and angle/length were no more than 2‰ and 2.5‰°/mm, respectively, which can meet the precision requirements of profile application.

## 5. Conclusions

In this paper, the hot twist-stretch straightening process of the Ti-6Al-4V alloy U-shaped profile was optimized by the aspects of deformation behavior, numerical simulation, and verification experiment. The main conclusions are summarized as follows:

1. The stress relaxation behavior is affected by temperature and time. The stress gradually decreases with the increases in time, and finally tends to the relaxation limit stress. The higher the temperature is, the lower the relaxation limit stress. Based on the stress relaxation curves, the Arrhenius creep constitutive model was established and verified, which could accurately describe the creep behavior of profile.
2. The straightening temperature and holding time have significant influence on the straightening process of the profile while the effect of stretch strain was relatively small. The combination of a straightening temperature at 700 °C, a stretch strain of 1.5%, and a holding time of 600 s is the optimal process parameter. The deflection/length and angle/length of profile after straightening are 1.5‰ and 1.7‰°/mm, respectively.
3. The verification experiments were carried out through straightening processes optimized by the orthogonal experiment. The experiment results are consistent with simulation results, which verified the accuracy of the established numerical simulation model.

## Figures and Tables

**Table 1:** Chemical composition of Ti-6Al-4V alloy profile (wt%).

Al	V	Fe	C	H	O	N	Ti
6.17	4.12	0.036	0.0044	0.0048	0.12	0.0073	Balance

**Table 2:** Parameters of the Arrhenius constitutive model.

*T*/°C	*A*	*α*	*n*	*Q* (J/mol)
500	4.05 × 10^−4^	0.0023	5.07	21,450
600		0.0046	3.02	19,255
700		0.0155	1.26	21,998

**Table 3:** Factors and levels of the orthogonal experiment.

Factors	Symbol	1	2	3
Straightening temperature/°C	A	500	600	700
Stretch strain/%	B	1	1.5	2
Holding time/s	C	0	600	1200

**Table 4:** Results of the orthogonal experiment.

No.	Temperature/°C (A)	Stretch Strain/% (B)	Holding Time/s (C)	Deflection/mm	Angle/°
1	500	1	0	17.4	30.7
2	500	1.5	600	8.7	17.9
3	500	2	1200	5.8	11.6
4	600	1	600	6.4	15.5
5	600	1.5	1200	3.2	9.2
6	600	2	0	11.3	18.3
7	700	1	1200	2.4	2.7
8	700	1.5	0	6.8	15.2
9	700	2	600	2.9	3.1
X1	10.6/20.1	7.3/16.3	11.8/21.4	The average value of deflection and angle corresponding to each parameter (deflection mm/angle°)	
X2	7.0/14.3	6.2/14.1	6.0/12.1		
X3	4.0/7.0	6.7/11.0	3.8/7.8		
R	6.6/13.1	1.1/5.3	8.0/13.6	The variation range of deflection and angle corresponding to each parameter (deflection mm/angle°)	

**Table 5:** Results of verification experiments.

No.	Length/mm	Before Straightening		After Straightening	
		Deflection/mm	Angle/°	Deflection/mm	Angle/°
1	2350	12	35	2	3
2	2760	38	42	3	5

## Data Availability

The data presented in this study are available on request from the corresponding author.
